# Trends in psychotropic drug consumption among French military personnel during the COVID-19 epidemic

**DOI:** 10.1186/s12916-022-02497-9

**Published:** 2022-09-14

**Authors:** Marc-Antoine Sanchez, Basile Fuchs, Pascale Tubert-Bitter, Anne-Sophie Mariet, Fabrice Jollant, Aurélie Mayet, Catherine Quantin

**Affiliations:** 1 Information Systems and Digital Department, French Military Health Service, Saint-Mandé, France; 2 Université Paris-Saclay, UVSQ, Université Paris-Sud, Inserm, High-Dimensional Biostatistics for Drug Safety and Genomics, CESP, Villejuif, France; 3 Centre Hospitalo-Universitaire Cochin, Paris, Assistance Publique Des Hôpitaux de Paris, Paris, France; 4 Service de Biostatistiques Et d’Information Médicale (DIM), CHU Dijon Bourgogne, INSERM, Université de Bourgogne, CIC 1432, Module Épidémiologie Clinique, 21000 Dijon, France; 5 Department of Psychiatry and Psychotherapy, Jena University Hospital, Jena, Germany; 6 Université de Paris, Paris, France & GHU Paris Psychiatrie Et Neurosciences, Hôpital Sainte-Anne, CMME, Paris, France; 7 McGill Group for Suicide Studies, McGill University, Montréal, Canada; 8 Nîmes Academic Hospital (CHU), Nîmes, France; 9 Moods Team, INSERM UMR-1018, CESP, Le Kremlin-Bicêtre, France; 10 French Armed Forces Center for Epidemiology and Public Health (CESPA), French Military Health Service, Marseille, France; 11 INSERM-IRD-Aix-Marseille université – SESSTIM, Marseille, France

**Keywords:** COVID-19, Psychotropic drugs, Military personnel

## Abstract

**Background:**

The coronavirus disease (COVID-19) pandemic may have had significant mental health consequences for military personnel, which is a population already exposed to psychological stress. To assess the potential impact of the COVID-19 pandemic, we analyzed the dispensing of three classes of psychotropic drugs (anxiolytics, hypnotics, and antidepressants) among French military personnel.

**Methods:**

A retrospective analysis was conducted using the individualized medico-administrative data of persons insured by the National Military Social Security Fund from the National Health Data System. All active French military personnel aged 18–64 who received outpatient care and to whom drugs were dispensed between January 1, 2019, and April 30, 2021, were included from the French national health database. Rate ratios of dispensed anxiolytics, hypnotics and antidepressants (based on drug reimbursement) were estimated from negative binomial regressions before and after the start of the COVID-19 pandemic.

**Results:**

Three hundred eighty-one thousand seven hundred eleven individuals were included. Overall, 45,148 military personnel were reimbursed for anxiolytics, 10,637 for hypnotics, and 4328 for antidepressants. Drugs were dispensed at a higher rate in 2020 and 2021 than in 2019. There was a notable peak at the beginning of the first lockdown followed by a decrease limited to the duration of the first lockdown. During the first lockdown only, there were temporary phenomena including a brief increase in drug dispensing during the first week followed by a decrease during the rest of lockdown, possibly corresponding to a stocking-up effect. For the study period overall, while there was a significant downward trend in psychotropic drug dispensing before the occurrence of COVID-19 (*p* < 0.001), the pandemic period was associated with an increase in dispensed anxiolytics (rate ratio, 1.03; 95% CI, 1.02–1.04, *p* < 0.05), hypnotics (rate ratio, 1.13; 95% CI, 1.11–1.16, *p* < 0.001) and antidepressants (rate ratio, 1.12; 95% CI, 1.10–1.13, *p* < 0.001) in the military population.

**Conclusions:**

The COVID-19 pandemic has probably had a significant impact on the mental health of French military personnel, as suggested by the trends in dispensed psychotropic drugs. The implementation of mental health prevention measures should be investigated for this population.

**Supplementary Information:**

The online version contains supplementary material available at 10.1186/s12916-022-02497-9.

## Background

The COVID-19 epidemic had a significant impact on mental health worldwide [1]. In France, observational studies in the general population reported high levels of anxiety or depressive symptoms in 34% of individuals during the first wave [2], and 42.8% of students declared experiencing at least one symptom of psychological distress [3]. The causes seem to be multifactorial, with direct and indirect effects. Notably, the measures implemented to contain the epidemic facilitated social isolation [4, 5], which is a major stressor. Moreover, approximately 10% of people who recover from COVID-19 present post-acute psychic sequelae of SARS-CoV-2 infection (PASC) such as fatigue, insomnia, depression, or anxiety [6, 7]. Globally, the COVID-19 pandemic has contributed to a worsening of mental health not just for patients but for the entire population [2, 8–11].

In the present study, we focused on a specific French population, military personnel, for several reasons. First, this population has been particularly exposed to COVID-19 through military operations (for instance, in the Sahel and in the surveillance of public places) or during the “Resilience” operation organized to support the population during the COVID-19 crisis. Second, this population is regularly exposed to significant stress, resulting in severe mental health consequences [12, 13] and potential operational repercussions [14, 15]. The COVID-19 epidemic could be an additional stressor. Third, the health effects of COVID-19 were less severe than in the general population since there were lower rates of hospitalization and fewer serious cases [16–18]. The military population is selected on physical criteria and is younger and healthier, which could be a protective factor. Overall, even though the mental health of the military population must be carefully preserved, the impact of the epidemic on this specific group, which was both highly exposed to COVID-19 and stress, and at a lower risk of severe COVID-19, is unclear and has been poorly studied. It is thus essential to assess the impact of the current crisis on the mental health of military personnel, especially considering the considerable physical and psychological pressure they are exposed to.

To understand the overall impact of the COVID-19 epidemic and the associated health policies on the mental health of the military, we investigated trends in the psychotropic drugs dispensed to this population. The main objective of this study was thus to analyze the impact of the COVID-19 epidemic on the use of psychotropic medication in the French military population since the beginning of the epidemic. Based on the available literature, we hypothesized that the sustained exposure to stress during the epidemic would have increased the consumption of psychotropic drugs, notably the most commonly prescribed classes, specifically anxiolytics, hypnotics, and antidepressants.

## Methods

### Study design and participants

A retrospective cohort analysis was conducted. All active French military personnel aged 18 to 64 years who received outpatient care requiring reimbursement between January 1, 2019, and April 30, 2021, were identified in the database.

**Fig. 1:**
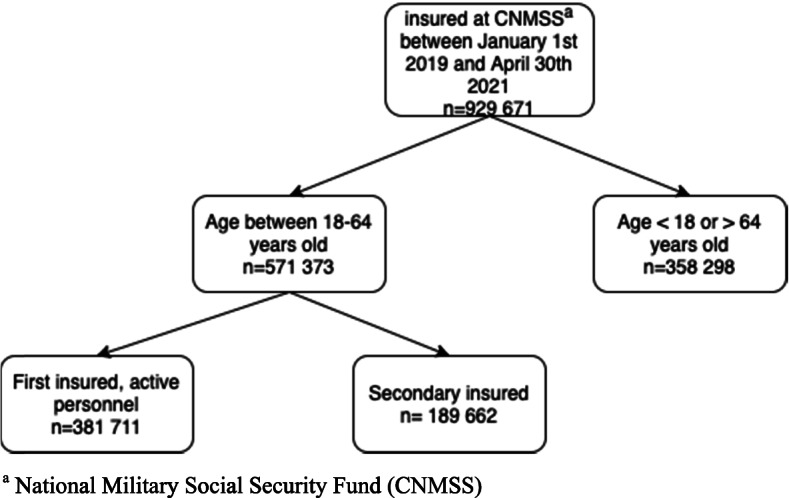
Flowchart for the identification of the studied population in the SNDS database

The selection was conducted as followed: (1) individuals affiliated to the National Military Social Security Fund (CNMSS), which is compulsory for military personnel; (2) aged 18 to 64 years (which is the age limit for active military personnel in France); (3) being the principal beneficiary of the health insurance; and (4) being an active service member, which means military personnel currently in service (Fig. 1). All individuals who met these criteria were enrolled in the study, whether at the beginning of the 2019 period or at the end in 2021. All included individuals were followed until the end of the study period in 2021.

### Data

The individualized medico-administrative data were extracted from the National Health Data System (SNDS) [19]. This database contains all the medical consumption or dispensing data for outpatient care, i.e., claims data (consultations, prescribed drugs, medical procedures, etc.) [20], for each person insured by the CNMSS. Drugs consumed during a hospital stay are not recorded in the SNDS.

### Independent variables

The impact of COVID-19 on psychotropic drug dispensing may have differed between the first weeks of the epidemic (associated with a lockdown in most countries) and later periods. For instance, stable or lower use of antidepressants and benzodiazepines were observed during the first 13 weeks of the epidemic in the USA [21]. It is therefore important to differentiate the short-term and long-term effects of the epidemic.

The temporal variable, “time,” was measured in weeks from January 2019 to April 2021 and coded week 1 to week 124. The start of the COVID-19 epidemic was a priori defined as week 4 of 2020, corresponding to the first official French case on January 24, 2020. The “covid” variable was coded 1 from the start of the epidemic, and 0 otherwise. The “time_after” variable, measured in weeks and coded 0 before the epidemic and 1 until 67, represents the time-dependent effect due to the epidemic. Sex was taken into account in the regression models. Age was coded in three categories: 18–25, 26–45, and 46–64 years.

### Outcome measures

We collected data relative to the number of reimbursed packs of different drugs per week during the COVID-19 crisis, in particular psychotropic medications, known as “support” drugs or drugs of interest to the French military, between 2019 and 2021.

We identified the three main psychotropic drug codes in the SNDS database using Anatomical Therapeutic Chemical (ATC) classification: anxiolytics (N05B), hypnotics (N05C), and antidepressants (N06A). For each category, the number of claims per week was collected over the study period, and rates were counted per week.

### Statistical analysis

We performed negative binomial interrupted time series (ITS) regressions [22] to study outcome variables before and during the COVID-19 epidemic using previously defined variables. We then estimated the corresponding rate ratios with their 95% CI (CI). The pre-epidemic period spans from January 1, 2019, to January 23, 2020, and the epidemic period from January 24, 2020, to April 30, 2021. To describe trends, segmented regressions were performed with a breakpoint on January 24. All time variables are described by week. Age category and sex were included in models as adjustment variables. Sensitivity analyses were performed to take into account a seasonal effect given the nature of the data used: the seasons considered were winter, spring, summer, and autumn.

During the epidemic period, we also identified 3 phases: (1) the first lockdown, extending from week 12 to week 19 (from March 17 to May 10, 2020), (2) the second lockdown between weeks 44 and 51 (October 30 to December 15, 2020), and (3) the last period (curfew) from weeks 13 to 17 (April 3 to May 3, 2021).

SAS Enterprise Guide software, version 7.1 (SAS Institute Inc.) was used for the descriptive analyses. The analytical part of the study was carried out using R 2021.09.1 with the “mfx” package.

### Ethics

The study was reported to the relevant authorities within the French Military Health Service. The French Military Health Service has permanent access to this database, made possible by the monitoring of specific training for the benefit of doctors and statisticians in charge of the analyses. This data is delivered thanks to an official French decree (*Décret n°2021–848 du 28 juin 2021, relative au traitement de données à caractère personnel “système national des données de santé”*)*.* All data were anonymous. No individual consent was necessary.

## Results

### Population

A total of 381,711 individuals were identified in the database as having active military status in 2019, being aged 18 to 64, and considered as the primary beneficiary (Fig. 1). The most frequent age group was 26–45 years. The proportion of consumers was approximately one-third females and two-thirds males, regardless of the category of the drug studied (Table 1).

**Table 1:** Main characteristics of the studied population in 2019, and psychotropic drug consumption between 2019 and 2021

	Overall^a^	Anxiolytics^b^ *N* (% of population)	Hypnotics^b^ *N* (% of population)	Antidepressants^b^ *N* (% of population)
Sex^a^				
Male	314,436	32,604 (10.4)	7763 (2.5)	3464 (1.1)
Female	67,661	12,544 (18.5)	2874 (4.2)	864 (1.3)
Age (years)^a^				
18–25	91,426	6628 (7.2)	1147 (1.3)	641 (0.7)
26–45	216,273	27,076 (12.5)	6039 (2.8)	2713 (1.3)
> 45	74,398	11,444 (15.4)	3451 (4.6)	974 (1.3)

^a^Description of the entire study cohort

^b^Global drug dispensing between January 1, 2019 and April, 30, 2021

### Dispensing of psychotropic medication

There was a general effect on the dispensing of all psychotropic categories from the start of the epidemic to the end of the studied period as compared with the pre-epidemic period.

**Fig. 2:**
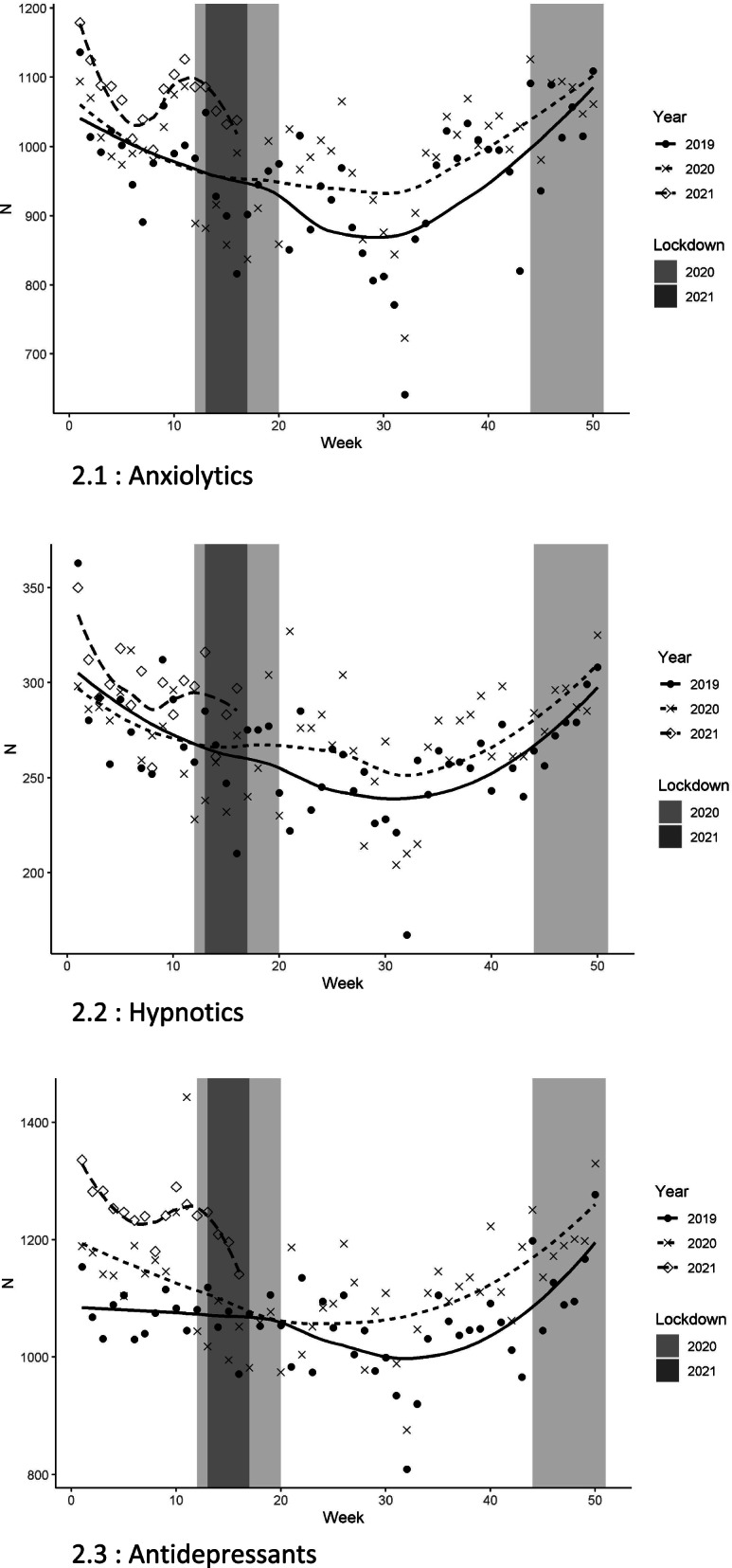
Psychotropic drug dispensing (anxiolytics, hypnotics, antidepressants) by week between January 1, 2019, and April 30, 2021, among the French military personnel, and lockdown periods (in grey). **2.1** Anxiolytics. **2.2** Hypnotics. **2.3** Antidepressants

A detailed examination revealed a number of fluctuations over the epidemic period. First, there was a brief upward peak in the reimbursement of antidepressants around the start of the first lockdown (weeks 10 to 12) (Fig. 2). Then during the rest of the first lockdown (weeks 11 to 19), we observed a temporary decrease in the reimbursement of anxiolytics and hypnotics compared to the previous year during the same period. Finally, starting at the end of the first lockdown (week 20), and regardless of the drug considered or the following lockdown periods, there was an increase in consumption that was maintained during the rest of 2020 and the first months of 2021 when compared to the same periods in 2019.

Table 2 shows the results of the modeling using interrupted time series. The adjusted model made it possible to highlight the specific effect of the epidemic on drug consumption by controlling for spontaneous changes in consumption over time. We observed (i) a downward trend in psychotropic consumption before the occurrence of COVID-19; (ii) a drop at the onset of the epidemic in France, followed by (iii) a progressive, slow and constant increase for each drug category. The rate ratios were 1.03 (95% CI, 1.02–1.04, *p* < 0.05) for anxiolytics, 1.13 (95% CI, 1.11–1.16, *p* < 0.001) for hypnotics, and 1.12 (95% CI, 1.10–1.13, *p* < 0.001) for antidepressants.

**Table 2:** Rate ratios for the dispensing of the three psychotropic drug categories between January 1, 2019, and April 30, 2021, among French military personnel, considering that the epidemic breakpoint date was January 24, 2020

	Anxiolytics (unadjusted)	Anxiolytics (adjusted)	Hypnotics (unadjusted)	Hypnotics (adjusted)	Antidepressants (unadjusted)	Antidepressants (adjusted)
Time^a^	0.9989^***^	0.9997	0.9980***	0.9975***	0.9994^*^	0.9987***
	(0.0003)	(0.0003)	(0.0005)	(0.0005)	(0.0002)	(0.0003)
Covid^b^	1.0462^***^	1.0314^*^	1.0786^***^	1.1321^***^	1.0567^***^	1.1145^***^
	(0.0124)	(0.0129)	(0.0243)	(0.0264)	(0.0117)	(0.0129)
time_after^c^	1.0024^***^	1.0019^***^	1.0031^***^	1.0037^***^	1.0020^***^	1.0029^***^
	(0.0003)	(0.0003)	(0.0006)	(0.0006)	(0.0003)	(0.0003)
Male vs female		0.8227		0.1114^***^		0.9973
		(0.1567)		(0.0241)		(0.2051)
Age 18–25 vs 26–44		0.1372^***^		1.9922^*^		1.7655
		(0.0386)		(0.6082)		(0.5420)
Age > 45 vs 26–44		0.4248^***^		0.6151^**^		0.0774^***^
		(0.0790)		(0.1130)		(0.0136)
Number of observations	123	123	123	123	123	123
Log likelihood	-2476.46	-2448.12	-1037.74	-966.28	-2226.24	-2083.11
Deviance	3885.94	3829.27	1167.61	1024.68	3369.36	3083.11
AIC	4960.92	4910.25	2083.49	1946.56	4460.48	4180.22
BIC	4976.98	4934.75	2099.55	1971.06	4476.54	4204.72

^***^p < 0.001; ^**^p < 0.01; ^*^p < 0.05

^a^Measured in weeks from 24th January 2020 to April 2021 (from week 1 to 124)

^b^Coded 1 from the start of the epidemic, defined at 24th January 2020, 0 otherwise

^c^Measured in weeks, coded 0 before the start of the epidemic and equal to the number of weeks afterwards (from week 1 to 67)

Men used hypnotics less often than women (rate ratio = 0.11; 95% CI, 0.08–0.12, *p* < 0.001). The increase in dispensed anxiolytics was lower in the 26–45 age group and higher in the 45–64 age group when compared to the 18–25 age group (rate ratio = 0.42, 95% CI, 0.34–0.50, *p* < 0.001; rate ratio = 0.14, 95% CI, 0.10–0.17, respectively). The 18–25 age group consumed 2.1 times more hypnotics (*p* < 0.05) than the 26–45 age group. We observed a time-dependent effect, with an increase in dispensing over time for all three drugs (“time_after” variable).

**Fig. 3:**
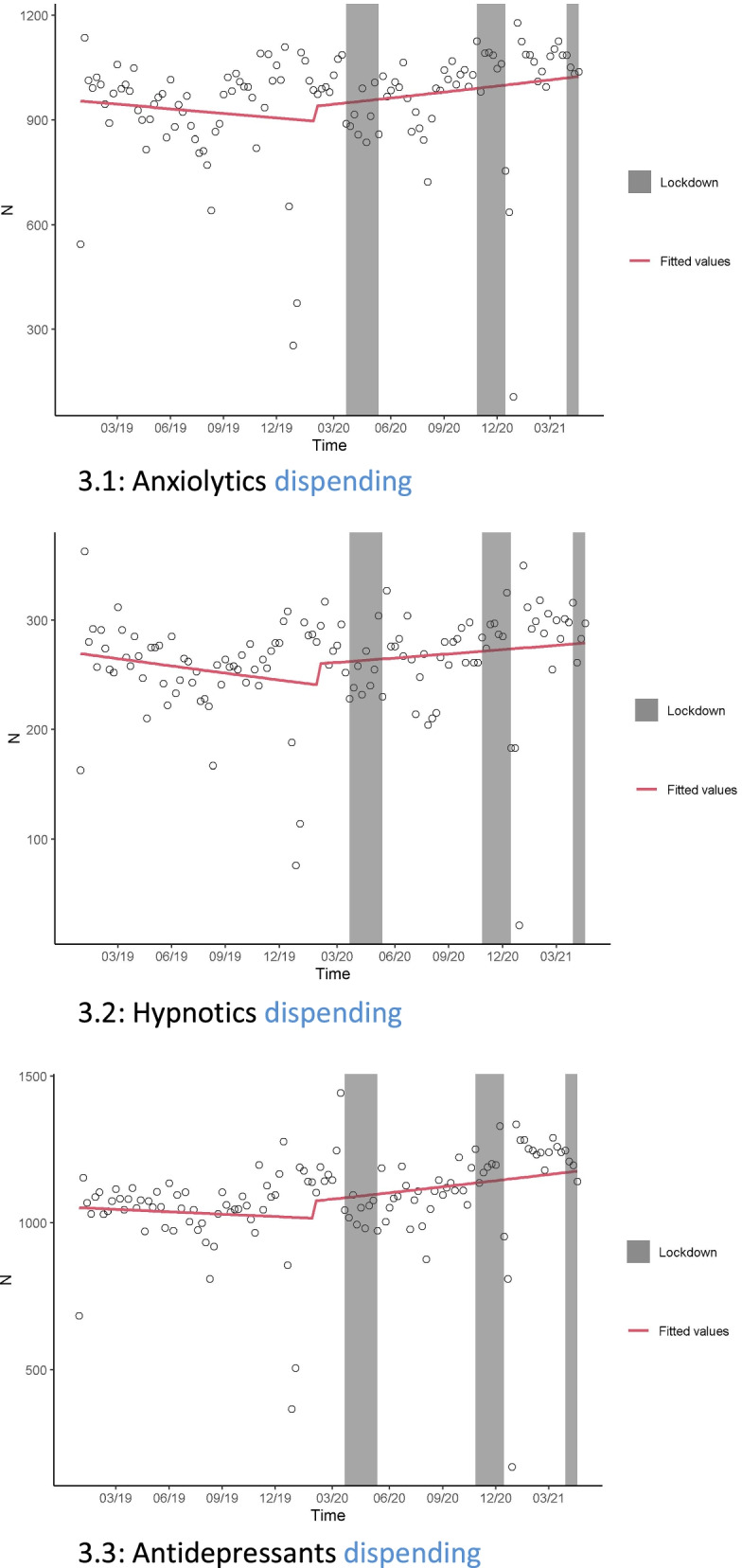
Modeling of psychotropic drug dispensing between January 1, 2019, and April 30, 2021, among the military personnel and restrictions periods, considering the date of epidemic breakpoint at 24 January 2020. **3.1** Anxiolytics dispensing. **3.2** Hypnotics dispensing. **3.3** Antidepressants dispensing

Figure 3 shows the overall effects. The breakpoint was the first case of COVID-19 in France, regardless of the therapeutic class studied. There was a positive slope which shows a lasting increase in consumption from the start of the epidemic onward (long-term effect). The results of the sensitivity analyses including a seasonal effect were consistent with those of the main analyses (Additional file 1: Table S1).

## Discussion

Our study reveals significant associations between changes in dispensed psychotropic drugs and the COVID-19 pandemic among French military personnel. In this study, which to our knowledge is the first to address the important subject of mental health in the military during the COVID-19 pandemic, we observed both temporary and overall effects. The first week first of the first lockdown was associated with a rapid rise in dispensed psychotropic drugs followed by a decrease during the rest of the lockdown. However, these effects were temporary, and the overall trends show an increase in the rate of dispensed psychotropic drugs since the start of the pandemic despite the downward trend in 2019. This overall increase suggests that the pandemic and associated containment measures had a significant negative impact on the mental health of military personnel, which remains a hypothesis to be confirmed by further studies. Over our study period, the rate ratio (2019 vs 2020–2021) increased by 3% for dispensed anxiolytics, by 12% for antidepressants, and by 13% for hypnotics. These increases are a key observation in our work. It could be hypothesized that fewer consultations with health professionals specializing in mental health may partially explain our results. In addition, due to the nature of the military activity, most military personnel have a job that is not compatible with telecommuting (collective sports and combat training in the field, on-site technical activities such as equipment maintenance), and some operations were maintained during the lockdowns despite the difficult conditions. On the contrary, some military personnel were not able to continue their usual activities, and the in-person training necessary for operational readiness was also impacted by the lockdowns, especially the first one. Overall, however, the lockdowns probably had the most marked effect on leisure time.

The initial transient phenomenon observed during the first lockdown may correspond to a “stocking-up” effect following the government’s announcement of the imminent lockdown. This effect was also observed in the general population [23], and it may have particularly involved people who were already under treatment. Certain measures were implemented to avoid a break in the continuity of treatment of patients suffering from chronic conditions and unable to consult as usual during the pandemic. For instance, the Ministry of Health rapidly authorized the delivery of treatments (especially antidepressants), from expired prescriptions, which may have affected patient behavior. It is worth noting that the stocking-up phenomenon was not observed during the second lockdown, which was not as strict [23]. Therefore, it may be that the sudden implementation of the first lockdown, which was a novelty for most people, and the fear of drug shortage led to the trend observed at the very start of the pandemic. The subsequent temporary decrease in the rates of psychotropic drug reimbursement during the same initial lockdown may correspond to the use of the drugs that had been purchased earlier and the limited access to general practitioners or specialists during that time. Although unlikely considering the increase in insomnia, depressive symptoms, and anxiety during this period in the general population [24], we cannot fully exclude that the decrease in drug dispensing may have been the result of a brief decrease in consumption.

The global upward trends in psychotropic consumption after the start of the COVID-19 epidemic have been verified in several studies [25], although not all [26]. The EPIPHARE study, a pharmaco-epidemiological study conducted by French public institutions and including data on the whole French population since 2018, reported changes in outpatient drug consumption and particularly a significant downward trend in the consumption of anxiolytics, hypnotics, and antidepressants until 2019, but which was interrupted in 2020 at the start of the pandemic [23]. While we observed an increase in antidepressant use among military personnel, who are generally healthy young adults, the generalization of these results should be considered with caution. For instance, in the general population, a temporary decrease in the reimbursement of antidepressants was also observed at the start of the pandemic, but it was followed by a rapid return to pre-crisis levels in August 2020 [27]. Therefore, the general population did not have the same increase in dispensed antidepressants that we observed among the military population. Other recent work from our team has underlined the challenge of drawing firm conclusions about the overall effects of the epidemic. For instance, our analysis of suicide attempts in the general population in France indicated that there was a decrease in self-harm hospitalizations both during the first lockdown [28] and during the second stage of the COVID-19 pandemic [29], except among teenage girls. It is therefore essential to take into account the specificities of the study population, and healthy young individuals from the general active population should be studied specifically before extrapolating our results.

It has been shown that the COVID-19 epidemic has directly or indirectly affected the mental health of various populations through different mechanisms, including increased stress at the family, professional or financial levels, the fear of contamination, or the effects of social isolation [30, 31]. Furthermore, mental health disorders due to the COVID-19 epidemic are risk factors for suicide ideation or self-harm [32]. The epidemic can thus be expected to result in longer-term effects on the consumption of healthcare for this selected population. Any interpretation of these effects must be done in light of all the health measures that were implemented in the military and in the general population to curb the epidemic. Moreover, the side effects of antipsychotics are not to be neglected. We can wonder whether even a minimal increase in the long-term consumption of these drugs could have long-term consequences (ischemic heart disease or acute stroke) [33, 34]. This remains, obviously, a hypothesis that will have to be the subject of further studies.

Further studies are needed to help to differentiate the effects of the factors involved, such as the epidemic itself, but also governmental measures (lockdowns and privation measures), taking into account occupational features and individual vulnerabilities as well as other potential confounding factors. Additionally, further studies should take into account occupational characteristics and individual vulnerabilities as well as other potential confounding factors that could not be taken into account in this study. It has been shown that specific mental preparation could help healthcare professionals suffer fewer mental health effects as a result of the pandemic. It is likely that a method of this type this could be applied successfully to military personnel as well [35].

The present study draws on comprehensive nationwide health care reimbursement data from the SNDS. It was therefore possible to precisely describe the changes in healthcare consumption of active military personnel in the civilian healthcare system thanks to the exhaustiveness of the database (not sample data).

Nevertheless, we recognize that there are limits to this work. First, though all drugs delivered by pharmacies are taken into account because they are systematically collected, we did not have access to data for drug consumption during hospital stays in the SNDS database. The SNDS database does not allow for the identification of military personnel in operation or the different armed forces (army, air force, navy, etc.), and the reimbursement database for outpatient care includes limited clinical data or data regarding lifestyle. However, the SNDS database includes information about certain comorbidities thanks to input from other databases including the long-term diseases database, the hospital information database (PMSI), and the drug dispensing database. Military personnel are subject to strict medical selection and surveillance. They are therefore rarely affected by the chronic comorbidities in the long-term diseases database because these are most often incompatible with the exercise of their functions. Additionally, while the database specified the delivery of drugs, actual consumption and thus compliance cannot be assessed, even though these are important factors for the study of psychiatric conditions. Due to their specific health system, French military personnel have access to a separate chain of care since their outpatient medical care can be dispensed within their home unit. Finally, from a statistical point of view, we used age groups in our analyses, which is standard practice, but we did not study within each group.

## Conclusions

This study shows that there were significant changes in the rate of psychotropic drugs dispensed among French military personnel during the COVID-19 epidemic, revealing the likely substantial mental health effects of the pandemic. Our findings suggest the need for enhanced vigilance considering the potential operational impact of psychiatric disorders in the military personnel. While the lockdown, particularly the first one, led to major changes in behavior in response to the crisis, the population appears to have rapidly adapted to the context. The French Military Health Service, which is responsible for the application of national and international recommendations, must be able to justify any modification to the nationally implemented prevention measures. In addition, military health services, both French and foreign, must be able to provide optimal support for their troops in times of crisis, and the ministries should better prepare their forces for the occurrence of such planetary events. To further this work, big data could be used to analyze care pathways and to detect trends that are not visible on a smaller scale.

## Supplementary Information

**Additional file 1:** Table S1. Rate ratios for the dispensing considering seasonal effect of the three psychotropic drug categories between January 1, 2019, and April 30, 2021, among French military personnel, considering that the epidemic breakpoint date was January 24, 2020.

## Data Availability

The database used in this study was transmitted by the National Health Insurance Fund -CNAM (Caisse nationale de l'assurance maladie) (responsible for the extraction of SNDS data). The use of these data by our department was approved by the National Committee for data protection. We are not allowed to transmit these data. Data are available for researchers who meet the criteria for access to these French data from the National Health Insurance Fund: training that opens a personal accreditation, approval of the protocol by required authorities (Expert Committee to researches, studies, and evaluations in the health field-CEREES, and National Committee for data protection-CNIL). Contact: Caisse Nationale de l’Assurance Maladie, 50 Avenue du Professeur André Lemierre, 75,020 Paris https://www.ameli.fr/assure/adresses-et-contacts

## Abbreviations

ATC: Anatomical Therapeutic Chemical
CI: Confidence intervals
CNMSS: National Military Social Security Fund
COVID-19: Coronavirus disease 2019
ITS: Interrupted time series
PASC: Post-acute psychic sequelae of SARS-CoV-2
PMSI: Hospital information database
SNDS: National Health Data System
