# Supplementary material for: Trends in psychotropic drug consumption among French military personnel during the COVID-19 epidemic
Source: BMC Med. 2022 Sep 14;20:306. doi: 10.1186/s12916-022-02497-9 (PMC9470234; doi:10.1186/s12916-022-02497-9)
Supplement: Supplementary file 1 — Additional file 1: Table S1. Rate ratios for the dispensing considering seasonal effect of the three psychotropic drug categories between January 1, 2019, and April 30, 2021, among French military personnel, considering that the epidemic breakpoint date was January 24, 2020. [file 12916_2022_2497_MOESM1_ESM.docx]

|  | Anxiolytics  (principal) | Anxiolytics  (season) | Hypnotics  (principal) | Hypnotics  (season) | Antidepressants  (principal) | Antidepressants  (season) |
| --- | --- | --- | --- | --- | --- | --- |
| Time ^a^ | 0·9989^***^ | 0·9982^***^ | 0·9980^***^ | 0·9977^***^ | 0·9994^*^ | 0·9993^***^ |
|  | (0·0003) | (0·0003) | (0·0005) | (0·0005) | (0·0002) | (0·0002) |
| Covid^b^ | 1·0462^***^ | 1·0708^***^ | 1·0786^***^ | 1·0960^***^ | 1·0567^***^ | 1·0590^**^ |
|  | (0·0124) | (0·0127) | (0·0243) | (0·0241) | (0·0117) | (0·0119) |
| time_after^c^ | 1·0024^***^ | 1·0028^***^ | 1·0031^***^ | 1·0030^***^ | 1·0020^***^ | 1·0019^***^ |
|  | (0·0003) | (0·0003) | (0·0006) | (0·0007) | (0·0003) | (0·0003) |
| Autumn |  | 1·1392^***^ |  | 1·1022^***^ |  | 1·0664^***^ |
|  |  | (0·0009) |  | (0·0178) |  | (0·0087) |
| Winter |  | 1·0226^**^ |  | 1·0527^**^ |  | 1·0190^*^ |
|  |  | (0·0009) |  | (0·0165) |  | (0·0081) |
| Spring |  | 1·0714^***^ |  | 1·0715^***^ |  | 1·0562^***^ |
|  |  | (0·0009) |  | (0·0164) |  | (0·0081) |
| Number of observations | 123 | 123 | 123 | 123 | 123 | 123 |
| Log Likelihood | -2476·46 | -2349·65 | -1037·74 | -1021·12 | -2226·24 | -2185·06 |
| Deviance | 3885·94 | 3632·32 | 1167·61 | 1134.37 | 3369·36 | 3287·01 |
| AIC | 4960·92 | 4713·30 | 2083·49 | 2056·24 | 4460·48 | 4384·12 |
| BIC | 4976·98 | 4732·99 | 2099·55 | 2075·93 | 4476·54 | 4403·81 |
| ^***^p < 0·001; ^**^p < 0·01; ^*^p < 0·05  ^a^ measured in weeks from 24th January 2020 to April 2021 (from week 1 to 124)  ^b^ coded 1 from the start of the epidemic, defined at 24th January 2020, 0 otherwise  ^c^ measured in weeks, coded 0 before the start of the epidemic and equal to the number of weeks afterwards (from week 1 to 67) | | | | | | |

**Supplementary file :**

Table S1 : Rate ratios for the dispensing considering seasonal effect of the three psychotropic drug categories between January 1, 2019, and April 30, 2021, among French military personnel, considering that the epidemic breakpoint date was January 24, 2020.
